# The Spleen: A Hub Connecting Nervous and Immune Systems in Cardiovascular and Metabolic Diseases

**DOI:** 10.3390/ijms18061216

**Published:** 2017-06-07

**Authors:** Andrea Lori, Marialuisa Perrotta, Giuseppe Lembo, Daniela Carnevale

**Affiliations:** 1 Department of Angiocardioneurology and Translational Medicine, IRCCS Neuromed, 86077 Pozzilli, Italy; lori.andrea.1985@gmail.com (A.L.); mlsperrotta@gmail.com (M.P.); 2 Department of Molecular Medicine, “Sapienza” University of Rome, 00161 Rome, Italy

**Keywords:** obesity, hypertension, splenic immune system, neuroimmune interactions, autonomic nervous system

## Abstract

Metabolic disorders have been identified as major health problems affecting a large portion of the world population. In addition, obesity and insulin resistance are principal risk factors for the development of cardiovascular diseases. Altered immune responses are common features of both hypertension and obesity and, moreover, the involvement of the nervous system in the modulation of immune system is gaining even more attention in both pathophysiological contexts. For these reasons, during the last decades, researches focused their efforts on the comprehension of the molecular mechanisms connecting immune system to cardiovascular and metabolic diseases. On the other hand, it has been reported that in these pathological conditions, central neural pathways modulate the activity of the peripheral nervous system, which is strongly involved in onset and progression of the disease. It is interesting to notice that neural reflex can also participate in the modulation of immune functions. In this scenario, the spleen becomes the crucial hub allowing the interaction of different systems differently involved in metabolic and cardiovascular diseases. Here, we summarize the major findings that dissect the role of the immune system in disorders related to metabolic and cardiovascular dysfunctions, and how this could also be influenced by neural reflexes.

## 1. Introduction

**Figure 1:**
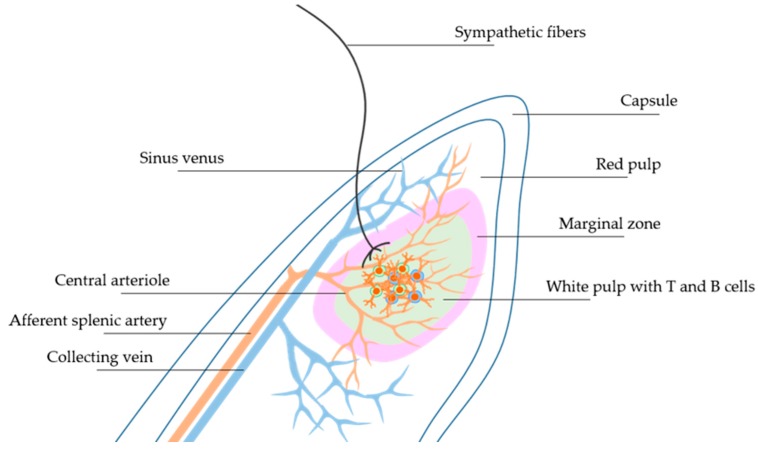
Schematic description of the architectural organization of splenic areas containing various immune cells: White Pulp (WP) and Red Pulp (RP) with Sympathetic nerve fibers innervating Marginal Zones (MZ).

Despite initially having been depicted simply as a blood filter, the spleen is gaining more attention for the pivotal role played in modulating immune responses [1]. The spleen is enclosed by an elastic and fibrous capsule with emerging trabeculae directed toward the internal parenchyma, ensuring the stability and maintenance of a well-organized structure [2,3]. The central arterioles that depart from the splenic artery branch in the trabeculae to subsequently dive in the White Pulp (WP) area, which contains B cells in the follicles and T cells. At this point, blood flows through the Marginal Zone (MZ) that surrounds the WP, toward the Red Pulp (RP) area, where blood is directed to the venous sinuses (Figure 1). Then, blood flows again through the trabecular area and is conveyed to the splenic vein. The splenic RP oversees the blood filtration process, in which resident macrophages phagocyte old erythrocytes, activate response to bacterial infiltration and allow iron recycling [1].

Another important activity that takes place in the RP is the production of antibodies. Indeed, plasmablasts differentiate in response to a specific antigen within the WP. Plasmablasts show enhanced production of CXC-chemokine receptor 4 (CXCR4), which specifically binds CXC-chemokine ligand 12 (CXCL12), which is produced in large quantities in the RP and is responsible for the migration and maintenance of cells in that splenic zone. This migration is also permitted due to the simultaneous repression of CXCR5 and CCR7 receptors on plasmablasts and lower response to the chemokines responsible for the homing in the WP follicles and T cell zone [3,4].

Splenic WP is a lymphoid area in which the specific B cell receptor CXCR5 is responsible for the organization of B cell follicles, where B cells are differentiated. T cells and Dendritic Cells (DCs) expressing the CCR7 receptor are attracted and confined in the T cell zone by the CC-chemokine ligands CCL19 and CCL21 [3,5]. Mutations that abolish the expression of these chemokines and receptors determine a completely aberrant distribution of the immune cells in the correct splenic districts [5].

Finally, the MZ, separating White from RPs, represents a connection between circulating blood and immune cells. This architecture allows B cells to defend the organism from blood-borne pathogens, mainly generating different lines of immunoglobulins (IgA, IgM and IgG) [6]. Alternatively, MZ B cells are able to activate CD4+ T cells to become antigen presenting cells (APCs) [7]. Populations of immune cells present in the MZ are not limited to B cells. Indeed, subsets of Neutrophils [6], Dendritic Cells [8] and Macrophages [9] can be found in the same area of the spleen. In order to maintain the correct separation between the MZ populations and the WP and RP immune cell populations, a specific pattern of surface membrane receptors and a different expression of chemokines are needed [10].

**Figure 2:**
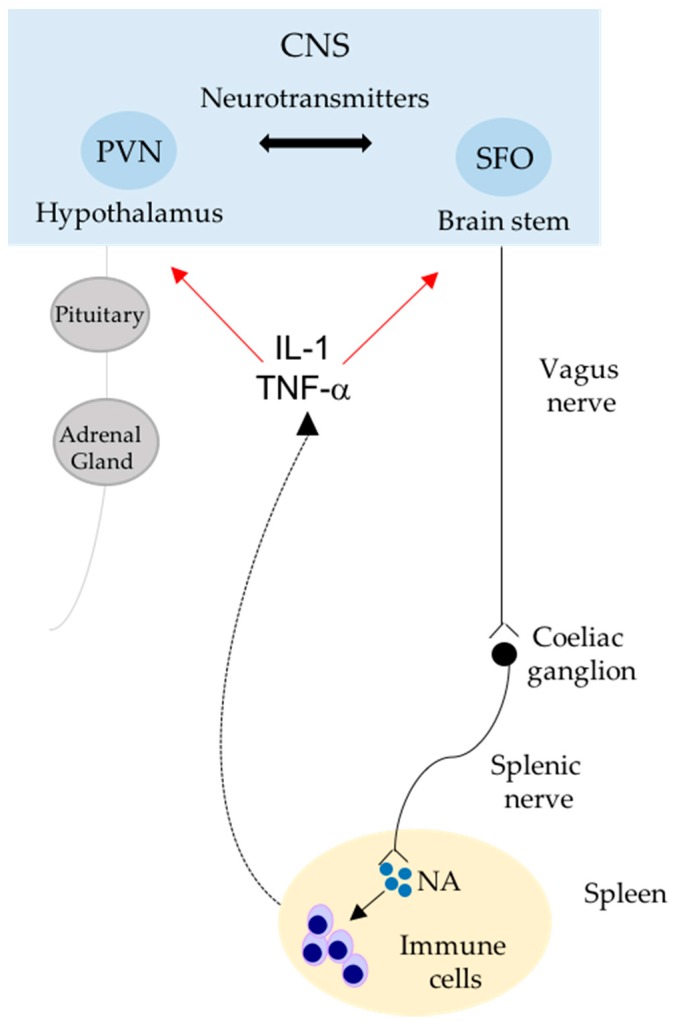
Bi-directional communication between the nervous and immune system. The scheme shows an integrative interface between the sympathetic nervous system (SNS), the hypothalamic-pituitary-adrenal (HPA) axis and the splenic immune reservoir. The SNS modulates immune cell activity through noradrenaline (NA) released in the spleen; and, in turn, the SNS activity is regulated by IL-1 and TNF-α cytokines, produced by splenic immune cells in a feedback loop manner. Paraventricular nucleus (PVN); subfornical organ (SFO); central nervous system (CNS). Double arrow: neuronal projections; red arrow: cytokine influence; black arrow: activation; dotted arrow: production.

In the last decades of the past century, researchers focused their attention on finding the basis of the immune system’s innervation, revealing that primary and secondary lymphoid organs receive fibers from the autonomic nervous system [11,12,13,14]. In particular, a variety of experimental approaches has been developed to deepen our understanding of the nature of splenic innervation [13]. It has been widely described that sympathetic nervous system (SNS) fibers densely innervate the spleen [15], and their synaptic endings are in close contact with immune cells [16,17,18]. Released neurotransmitters reach the surface of macrophages and/or lymphocytes, interacting with their specific receptors and modulating immune cells responses (i.e., inhibiting or enhancing expression of different cytokines) [12,14,19]. These properties characterize the neuro-immune communication. Once activated, immune cells produce and secrete a variety of pro- or anti-inflammatory mediators, which provide a tuned immune cell response. Importantly, neuro-immune communications should not be considered a one-way pathway from neurons to immune cells. It has been described that a bi-directional communication does exist [20,21]. Not only neurotransmitters released by neuronal terminations influence immune cells activity, but, in turn, cytokines released from immune cells can influence functions of neurons via cognate receptors on the neuronal cell surface [22,23,24]. Indeed, T cells are able to modulate noradrenaline (NA) pathways, reducing sympathetic fibers in the spleen and affecting the pituitary-adrenal axis in central nervous system (CNS) through diminished hypothalamic NA concentrations [24] (Figure 2).

In this view, the spleen appears as a crucial lymphoid organ, housing different populations of monocytes and macrophages, with B and T lymphocytes allowing both innate and adaptive immune responses. Besides this, the neuro-immune interaction provides a fundamental connection between the resident immune cells and the adjacent fibers of the autonomic nervous system innervating the spleen.

## 2. Immune System and Metabolic Disease: Meta-Inflammation, Obesity and T2D, Adipokines

Obesity is a major health problem with growing incidence, especially in childhood, increasing the possibility of developing, in adulthood, metabolic or cardiovascular co-morbidities such as type II diabetes (T2D), atherosclerosis and hypertension [25,26,27]. Excessive nutrient intake and low energy consumption with a consequential increase in adipose tissue size are conditions that could lead to obesity. In parallel with the incidence of obesity, patients frequently develop insulin resistance (IR), a leading cause of type II diabetes (T2D). It is widely accepted that enhanced inflammation is a common and central feature in metabolic syndrome. However, in contrast to the classic short-term inflammatory response to infectious agents that is typically followed by a return to baseline conditions, metabolic syndrome manifests alternative features of inflammation. A long-term and low-grade inflammation characterizes the so-called “meta-inflammation” [28]. Derangement in the immune cells homeostasis toward a steady increase in pro-inflammatory response is a key aspect that leads to the onset of such pathophysiological conditions [29].

Studies conducted both in humans and animal models outline that obesity-induced inflammation occurs in many organs involved in energy homeostasis: liver [30], muscle [31,32], pancreas [33], and adipose tissue [34]. Metabolic dysfunctions affect not only peripheral organs but also the CNS, especially in those hypothalamic areas involved in the control of energy balance, such as the arcuate nucleus (ARC), the ventromedial, lateral and paraventricular hypothalamic nuclei [35,36,37].

Despite all the biological aspects connecting obesity with related diseases that have not already been discovered, several studies have emphasized the relationship between obesity-induced pro-inflammatory response and the onset of metabolic syndrome. In order to investigate the causes of obesity-induced inflammation and IR, researchers at first focused their attention on fat pad. Lean adipose tissue is normally populated by different innate and adaptive immune cells, designated to maintain an anti-inflammatory phenotype and tissue integrity [38]. In addition, they have been shown to secrete anti-inflammatory cytokines with the aim of promoting insulin sensitivity, lipolysis, mitochondrial respiratory capacity, oxygen consumption and inhibiting lipogenesis [39].

Conversely, obesity provides conditions under which adipose tissue changes, altering the functions of immune cells, which contributes to the derangement of a balanced type-1 and type-2 immunity [29]. Importantly, murine models and human studies demonstrate that the quantity of adipose tissue macrophages (ATMs) bears a close relationship with the amount of adipose tissue itself.

Specifically, ATMs in lean mice account only for 10% of cells in the adipose tissue, and are evenly dispersed. In the lean state, macrophages express specific anti-inflammatory markers, such as MGL1 (macrophage galactose *N*-acetyl-galactosamine specific lectine 1), Arginase1, CD301, CD206 and CD163 on their cellular surface [40,41,42]; and the anti-inflammatory environment is maintained through the secretion of anti-inflammatory cytokines IL-4 (produced also by eosinophils), IL-13 and IL-10, with CD4+Foxp3+ regulatory T cells improving insulin sensitivity [29,40,42,43]. Since the controlled expression and secretion of anti-inflammatory mediators is pivotal to maintaining basal immune homeostasis in adipose tissue, ATMs and regulatory T cells are not the only cell types homing in on the adipose tissue of lean mice. Recently, a population of innate-like B, similar to B-1a and B10 B cells, has been identified in the visceral adipose tissue (VAT) of lean mice [44]. These cells, producing high amounts of IL-10, are critical players in maintaining the correct balance of immune cell populations toward an anti-inflammatory environment. The surgical procedure of splenectomy provided important evidence that the spleen is involved in the modulation of immune balance, being the source of innate-like B cells [44]. VAT of high fat diet (HFD)-fed splenectomized mice indeed showed reduced levels of innate-like B cells compared to sham mice, whereas the number of other lymphocytes was comparable [44]. In agreement, adoptive transfer of innate-like B cells in obese mice ameliorated both insulin sensitivity and glucose tolerance [44]. Others have discovered a different B cell population, called adipose natural regulatory B cells (Breg) [45]. This subset has been found to be crucial in decreasing CD44 and interferon-γ (IFN-γ) production in CD8+ T cells within visceral and subcutaneous fat depots. In addition, these cells were shown to improve IR by ameliorating tissue homeostasis through the constitutive production of IL-10, both in mouse models and in humans [45]. Through experiments of adoptive transfer of adipose tissue B cells in diet-induced obesity (DIO) mice, it has been demonstrated that Breg cells of non-splenic origin were found in adipose tissue, pointing out the possible presence of other routes of recruitment [44]. In a different work, it was shown that B cells are involved in the onset of inflammation and IR upon HFD feeding, promoting the release of CD4+ and CD8+ T cells cytokines and IgG2c pathogenic antibodies both in mouse models and in humans [46]. In addition, the IgGs produced enhanced the switch of macrophages toward pro-inflammatory phenotypes, thus contributing to development of metabolic disorders [46]. This evidence highlights the fact that the surveillance of systemic immune homeostasis is granted by specific populations of B and T cells, which in part are provided by a secondary lymphoid organ, the spleen, that preserves an anti-inflammatory immune environment. On the other hand, the existence of B cell subpopulations that expand, boosting the early phase of inflammation in obesity, demonstrates that B cells are also involved in the first stage of metabolic syndrome with the production of autoantibodies.

In obesity, the immune scenario completely changes through the massive production and secretion of chemoattractant molecules or pro-inflammatory cytokines. Adipocytes progressively increase in number and dimension. Macrophages with a pro-inflammatory phenotype massively infiltrate the adipose tissue and, along with resident ATMs, begin pathological proliferative processes. The percentage of ATMs rises to 40–50%, and they are specifically detected surrounding the necrotic and enlarged adipocytes, forming typical “crown-like structures” (CLSs) [40,47,48,49]. ATMs confined in these structures are characterized by membrane expression of specific pro-inflammatory markers, such as CD11c, TLR4, and a reduced production of anti-inflammatory cytokine IL-10 [40]. In addition, ATMs (CD11c+CD206−) stimulated with pro-inflammatory cytokines such as IFN-γ, in turn produce abundant factors including TNF-α, IL-1β, IL-6 and nitric oxide (NO) [46,50]. A different study with HFD-fed mice showed that more pro-inflammatory F4/80+CD11c+ macrophages were recruited in adipose tissue of obese mice compared with lean mice and, conversely, HFD-fed C–C motif chemokine receptor 2 (CCR2) KO mice showed a decreased content of the same population of immune cells, thus suggesting that infiltrating cells were recruited through a mechanism mediated by the chemokine monocyte chemoattractant protein-1 (MCP1/CCL2) and CCR2 [38]. The progressive ATM accumulation in adipose tissue and the proportional increase in inflammatory mediator production give rise to a cascade of transcription factor activations and other pro-inflammatory cell recruitments, which dramatically and further strengthen the inflammatory response and progression of metabolic syndrome. Wu et al. demonstrated that, in obesity, the enrollment of splenic innate-like B cells, along with their anti-inflammatory activity, is not sufficient to counteract the rising inflammation and IR development [44]. With developing obesity, CD4+Foxp3+ regulatory T cells are no longer able to counteract pro-inflammatory IFN-γ secreting CD8+ and CD4+ T cells [28]. Natural killer (NK) cells in visceral adipose tissue are stimulated, in obesity, to produce IFN-γ, participating to the inflammatory response. Mice that were deficient of NK cells, adipocyte NK-activation receptors (NCR-1) or IFN-γ, demonstrated reduced recruitment of pro-inflammatory macrophages in VAT and ameliorated obesity-induced IR [51]. In contrast, IL-15-induced NK cells aggravated metabolic disease, favoring IR and adipose tissue inflammation [52]. As a further mechanism modulating the immune response involved in metabolic disease, it has been shown that adipose tissue releases free fatty acids (FFAs), which enhance inflammation trough Toll-like receptor 4 (TLR4) via Fetuin-A (FET-A) and the consequent activation of Nuclear factor KB (NF-KB) induced IR. In addition, it has been reported that expression of both FetA and TLR4 is essential for development of IR in the presence of FFAs [53].

Taken together, these findings suggest that the immune system plays a fundamental role in the maintenance of the homeostatic physiological environment. However, in response to specific stimuli, a dysregulation of the immune system may become a principal actor in the development of metabolic syndrome.

## 3. Metabolic Disease as a Risk Factor in CVD and Hypertension

In the contest of obesity, an abnormal increase of adipose tissue has been correlated with a concomitant increased risk of cardiovascular disease (CVD) incidence. Moreover, the contribution of different adipose depots in the development of obesity-related diseases has been investigated. Different studies on humans and mouse models, in fact, ascribe to VAT—and not to Subcutaneous Adipose Tissue (SAT)—a predominant role in IR and CVD [54,55,56].

As described above, adipose tissue can not only be considered as a place where energy is preserved but, notably, it also has endocrine activity producing a wide range of molecules involved in metabolic processes [37]. The proteins that exert immuno-metabolic or cardiovascular functions, and are produced and secreted by adipose tissue, are also known as adipokines. These molecules carry out their effects modulating appetite and energy expenditure, insulin and glucose metabolism, as well as blood pressure, reaching the CNS and acting on neurons in specific brain areas. On this note, the melanocortin system resides in the hypothalamus and acts as a fulcrum of energy balance regulation [57]. Two different populations of neurons participate with opposite roles in the control of energy balance: on one hand, the orexigenic agouti-related peptide (AgRP) and neuropeptide (NPY) neurons; on the other, the anorexigenic proopiomelanocortin (POMC) neurons, whose activity can be directly regulated by metabolic hormones [57] and inflammatory molecules [35].

As a consequence of obesity, an immune regulator as important as IKKβ/NF-κB is involved not only in meta-inflammation of peripheral tissues, but is also activated in the CNS, participating in hypothalamic inflammation and aggravating IR and Leptin resistance development [35]. Leptin has been one of the first and most-analyzed adipokines. It is a hormone specifically expressed by adipose tissue, and its production is proportional to the quantity of fat pad [58]. The main role exerted by Leptin is the regulation of energy balance, acting directly in the CNS, and being able to cross the blood brain barrier (BBB) [59,60]. Besides its effect on energy control, Leptin is involved in the CNS modulation of sympathetic nerve activity (SNA). Targeted deletion of the Leptin receptor (ObR), specifically in the ARC, blocks diet-induced increased renal sympathetic activity and blood pressure rise [61]. Another study links Leptin action to the circumventricular subfornical organ (SFO), highlighting that deletion of Leptin receptors (ObR) in the SFO did not affect food intake behavior in mice, but suppressed SNA [62]. Studies conducted in both humans [63,64,65,66] and DIO mouse model clearly showed that, during obesity, the higher level of Leptin concentration correlated with SNA in renal nerves and blood pressure increase [67,68]. Thus, it is clear that obesity-related adipokines sustain neuroinflammation affecting many brain areas and producing different deleterious consequences to systemic homeostasis beyond feeding behavior and energy imbalance.

To date, many studies have described that the modulation of renal sympathetic activity is strictly related to hypertension induced by several pathophysiological alterations, like obesity [69]. However, it has recently been uncovered that sympathetic outflow plays a crucial role in hypertension, even directly modulating immune function though a control of splenic sympathetic nerve activity [12,13]. With obesity being characterized by chronic low-grade inflammation, and at same recognized as a dominant risk factor for hypertension, studies aimed at discerning the possible role of the neural modulation of splenic immunity in affecting the metabolic and cardiovascular consequences of the disease are warranted.

## 4. Neural Regulation of Immunity in Hypertension

The fact that immune cells and the sympathetic nervous system (SNS) strongly contribute to hypertension is a well-consolidated notion but, more recently, it has also been highlighted that these two elements are strictly intertwined in the modulation of blood pressure regulation. By using RAG-1^−/−^ mice, lacking both B and T cells, it was firstly shown that lymphocytes, infiltrating tissues typically targeted by increased blood pressure levels, are essential for the onset of hypertension [70]. In the same experimental setting of Angiotensin (AngII)-induced hypertension, it has been demonstrated that the T cells found in target tissues as kidneys and vasculature originate in the spleen [12]. In particular, by taking advantage of mice differing for the leukocyte antigen CD45, expressed in the two isoforms CD45.1 and CD45.2 in two different strains, it was possible to generate chimeric animals by spleen transplantation in order to label T cells of splenic origin with the CD45.1 marker (CD45.1 donor mouse), differentiating them from those of the recipient mouse (CD45.2 positive). After infusion of AngII, T cells infiltrating aorta and kidneys were found to be positive for both CD45.1 and CD45.2 antigens, thus indicating that the hypertensive challenge recruited a movement of immune cells from the spleen to allow colonization of target organs [12]. However, whether an immune response originating in the spleen is also recruited in hypertension resulting from other causes, such as that reproduced with deoxycorticosterone acetate (DOCA)-salt or obesity murine models, is still unknown. Certainly, the recently discovered neural regulation of immunity during AngII-hypertensive challenge is suggestive that molecular mechanisms activated in the spleen to prime immunity could be shared by various stimuli capable of exerting a neurogenic action [12,13,71,72]. On this note, the fact that chronic hypertensive stimuli, such as AngII and DOCA-salt, cause an increase of blood pressure levels activating renin-angiotensin system (RAS) in the brain through the AngII type 1 receptors (AT1Rs) in the SFO is well known [72]. This observation fits well with more recent evidence that both AngII and DOCA-salt activate an increased sympathetic outflow in the spleen [13], suggesting that, overall, different hypertensive challenges share neurogenic mechanisms to prime the immune system through neuroimmune pathways. Conversely, to date, there is still no evidence of the possible involvement of this neuroimmune pathway in the development of arterial hypertension associated with metabolic syndrome.

It is interesting to note that Harlan and colleagues, some years ago, demonstrated that the intracerebroventricular (ICV) administration of Leptin in the ARC of mice was able to significantly increase the renal sympathetic nerve activity, leading to arterial hypertension [61]. In light of current knowledge of neuroimmune pathways that are activated by sympathetic outflow in the spleen and are crucial for the onset of hypertension, it could be envisaged that even metabolic stimuli could share similar neurogenic mechanisms coupled with immune priming to increase blood pressure [62].

On a different note, immunologists discovered for the first time that the autonomic nervous system has a critical role in modulating immune responses in infectious settings. Several years ago, Tracey and colleagues published an amazing work that provided the basis for unraveling a circuit that connects the brain to the spleen and is able to dampen exacerbated innate immune responses during endotoxemia [17]. In particular, they showed that the electrical stimulation of the vagus nerve was effective for hampering the excessive TNF-α production induced by Lipopolysaccharide (LPS) administration in mice [17]. It was also proved that this pathway was mediated by α7-nicotinic cholinergic receptors (α7nAChRs) expressed on macrophages in the spleen, supporting the existence of the so-called “cholinergic anti-inflammatory reflex” [17].

Some other evidence, showed that the peripheral administration of the acetylcholinesterase inhibitor galantamine, typically used for the treatment of neurological disorders [73,74], has an anti-inflammatory effect, reducing serum TNF levels in endotoxemic mice [74]. Surprisingly, a similar galantamine treatment was shown to ameliorate inflammation and obesity in mice, significantly improving blood glucose and IR [75]. Indeed, mice fed with HFD and concomitantly treated with chronic galantamine exhibited a reduction of circulatory cytokines and adipokines, specifically IL-6 and Leptin [75]. Overall, these reports suggest that further insight is required to understand how neuroimmune mechanisms modulate metabolic, and probably cardiovascular, dysfunction in obesity.

Adipose tissue growth is closely related to angiogenesis [76]. Besides its role in secreting adipokines, regulating feeding behavior, and modulating SNA, Leptin plays an important role in angiogenesis, too [76]. During obesity, fat depots also produce other molecules involved in angiogenesis, such as the Vascular Endothelial Growth Factor (VEGF) [76], or the Placental Growth Factor (PlGF) [77], which belong to the family of the Vascular Endothelial Growth Factors (VEGFs). These growth factors have been considered novel therapeutic targets for the treatment of human obesity, given results obtained in experimental models suggesting the protective roles of antiangiogenic molecules, highlighting their potential therapeutic application [76]. On a similar note, the more recent role proposed for PlGF as a modulator of inflammatory and immune responses in cardiovascular settings further suggests that its involvement in metabolic syndrome could deserve further investigation, being involved in roles other than angiogenesis [78,79,80]. Indeed, the fact that PlGF has also been shown to play a pivotal role in the onset of hypertension, by mediating neuro-immune interactions in the spleen [12], suggests the possibility that its implications in obesity could also be related to the cardiovascular consequences of metabolic alterations, as is the case for increased risk of hypertension itself. A report in the literature suggests that the inactivation of PlGF in a murine model of HFD-induced obesity leads to reduced adipose tissue growth with a concomitant reduction of angiogenesis [77]. In *ob/ob* mice, a murine model of obesity characterized by mutations in the gene responsible for the production of Leptin, the absence of PlGF significantly prevented weight gain [81]. However, as mentioned above, obesity is also strictly related to chronic inflammation and increased risk of developing cardiovascular disease [47,48], thus suggesting the need to explore possible roles of angiogenic growth factors in mediating the immune functions involved in obesity and related complications.

## 5. Conclusions

Obesity and related metabolic disorders are recognized as risk factors for the onset of cardiovascular diseases, and hypertension in particular. Both these conditions share complex and not-yet-completely-understood aspects. The immune system is not only the main player responsible for preserving the host from exogenous hostile infiltration, but increasing evidence sustains the idea that it is also involved in the regulation of multiple biological aspects. Derangements in the homeostasis of immune components are pivotal factors in the onset of metabolic diseases and end-organ damage in hypertension. Despite this, the origin and the activation pathways of infiltrating immune cells in a multifactorial disease, such as metabolic syndrome, have not been fully described yet. However, impaired activity of the autonomic nervous system has been deepened in response to several metabolites produced during obesity and insulin resistance. The recent discovery of a neuro-immune interaction that primes activation and migration of splenic immune cells has revealed that the nervous system modulates immunological responses evoked by hypertensive stimuli. Therefore, it can be hypothesized that the same neuro-immune communication in this secondary lymphoid organ contributes to the refueling of pathological immune cells in adipose tissue during metabolic disease.
